# Sustainable Development Goals and Physical Education. A Proposal for Practice-Based Models

**DOI:** 10.3390/ijerph18042129

**Published:** 2021-02-22

**Authors:** Salvador Baena-Morales, Daniel Jerez-Mayorga, Pedro Delgado-Floody, Jesús Martínez-Martínez

**Affiliations:** 1 Department of General and Specific Didactics, Faculty of Education, University of Alicante, 03690 Alicante, Spain; 2 Faculty of Rehabilitation Sciences, Universidad Andres Bello, 7591538 Santiago, Chile; daniel.jerez@unab.cl; 3 Department of Physical Education, Sports and Recreation, Universidad de La Frontera, 4811230 Temuco, Chile; pedro.delgado@ufrontera.cl; 4 Department of Physical Education, Artistic Education and Plastic, Faculty of Education, University of Castilla-La Mancha, 45071 Toledo, Spain; jesus.mmartinez@uclm.es

**Keywords:** sustainability development, physical education, physical activity, pedagogical research

## Abstract

The Sustainable Development Goals (SDGs) is a global strategy that aims to obtain a more equitable and just world. These objectives are organized into 17 SDGs, detailing 169 targets. Different international institutions have emphasized the relevance of education to developing citizens who contribute to achieving the SDGs for 2030. However, a review focused on physical education (PE) has yet to be performed. Therefore, the objective of this work is two-fold. First, to analyze and select the specific SDGs that can be implemented in the area of physical education. Second, to relate these specific goals to the different models based on physical education practices. This review showed how three institutional documents have previously related sport, physical exercise, and physical education to specific SDGs. Based on the search done, this review article selects those goals that could be integrated into the educational context through physical education. The bibliographic and critical analysis in this research shows that of the 169 specific goals proposed in the SDGs, only 24 could be worked on in physical education. Upon completion of the analysis, a proposal for the relationship between the practice-based models and these 24 goals is presented. The contributions made in this paper will allow teachers to establish links between PE sessions and SDGs while raising awareness to develop students who contribute to a more sustainable world.

## 1. Introduction

### 1.1. Agenda 2030 and Education

The growing global concern about caring for the planet and ensuring prosperity for all meant that by 2015, the United Nations established a series of global goals [1]. These objectives are grouped into 17 Sustainable Development Goals (SDGs), which in turn are divided into 169 specific targets that detail and specify each of the SDGs [2]. This proposal of global sustainability is framed under the concept “Agenda 2030” and emphasizes that all levels of society, both collective and individual, must contribute to achieving the SDGs [3]. In addition to focusing on ecology as a central axis, these objectives include additional problems such as circular production, waste generation, poverty or health and welfare, urban development or peace, among others [1]. In this way, many spheres of intervention will be covered, not only environmental but also economic, ethical, and sociological [4], with a clear purpose, such as balancing current development with future progress. However, although the SDGs are a proposal to be achieved in 15 years (2015–2030), some research has highlighted that the SDGs’ pace of achievement is not as fast as expected [5]. Therefore, all government and non-government institutions must collaborate to facilitate the acquisition of these goals [6].

Within these institutions, education should be considered a key factor in consolidating sustainable habits in future generations [7,8]. The United Nations Decade of Education for Sustainable Development (2005–2014) [9] already detailed this importance, pointing to integrating sustainable development actions into all aspects of education to promote changes in knowledge and attitudes towards sustainability. Therefore, at the institutional level, education, in all its amplitude, is one of the main axes on which to structure sustainability [10]. Lauder et al. [11] highlighted the importance of education in responding to the planet’s socio-environmental problems. This importance is recognized by Sachs [6], which highlighted how education was a key factor in achieving the longstanding proposal of the Millennium Goals.

Although education is addressed directly in SDG 4, “Quality Education,” United Nations Educational Scientific and Cultural Organization (UNESCO) [12] established the concept of Education for Sustainable Development (ESD), aimed at empowering students to make responsible decisions in pursuit of a just society, economic and environmental integrity in the present and future generations. It has been pointed out that teachers play a decisive role in ESD as those responsible for educating future generations [13]. However, the different institutions must clarify their goals, indicators, and concrete actions concerning the SDGs to develop results frameworks and collect data on the level of achievement [14].

### 1.2. Physical Education, Physical Activity, and Sport. A Key Tool in Obtaining the SDG

The study of sport and physical activity (PA) pertaining to the SDGs has been mainly carried out by three institutions. First, the Sixth International Conference of Ministers and Senior Officials Responsible for Physical Education and Sport (MINEPS VI) [15]. This Conference identified three broad areas of intervention that aimed to (i) develop an inclusive vision of access for all to the sport, physical education (PE) and PA, (ii) maximize sport’s contribution to sustainable development and peace, and (iii) protect the integrity of the sport. This paper reviews the various SDGs proposed to highlight those relevant to PA and sport (Table 1). However, the PE role in Agenda 2030 is not specifically set out. Parallel to MINEPS VI, the Commonwealth published a document where the contribution of sport to the SDGs was clarified [16]. This document had the peculiarity of establishing a series of indicators and achievements that will measure the contribution of the different areas associated with sport more objectively when developing the SDGs. At the same time, it served as a reference along with the indications presented at MINEPS VI, to publish a new report detailing what specific targets of each SDG could be worked on through sport [14], however, the specifics regarding PE were not detailed. 

Finally, in 2019, the Ibero-American Sports Council and the Ibero-American General Secretariat jointly published a report establishing sport as a tool for working towards sustainable development [17]. In this case, they selected those targets of the SDGs that could be worked on through the PE, sports practice, or sport as an institution while establishing a thorough analysis of how this relationship would occur (one-way or two-way) and proposed a series of strategies and partnerships for the promotion of sustainable development. This report’s main findings were that not all the specific goals for each objective have the same directionality and ramifications with sport, highlighting instead a direct relationship with eight of the 17 SDGs and 19 of the 169 specific targets (Table 1). Another example of sport and PA’s relationship with the SDGs is reflected in the narrative review of Day and Menhas [18]. However again, these authors cannot separate the relationship between PE and the specific goals proposed by UNESCO. Finally, other institutions such as the WHO have confirmed these relationships and shown the health, social, and economic benefits of contributing to 13 of the 17 SDGs, but do not specify concrete goals that could be worked on. Rather, these institutional proposals emphasize the unified analysis of the concept of sport, physical exercise, and PA, so much so that MINEPS VI [15] begins by considering this conceptual appreciation, “the term “sport” is used as a generic term that includes sport for all, physical play, leisure, dance, and organized, improvised, competitive, traditional and indigenous sports and games in their different manifestations” (p. 1). In contrast, the Commonwealth details the terminological differences between sport, organized sport, PA, physical exercise, PE, and quality PE [14]. However, despite recognizing these differences, the selection of targets from each SDG was perceived from a general perspective of sports. 

In short, it is of particular interest and necessity to establish meeting points to address the different challenges of the future from a multidisciplinary approach, and that is where the main objective of the study resides when establishing the possible relationships between PE, as a discipline that has among other curricular objectives PA in the natural environment as enjoyment and care of the environment, and the Sustainable Development Goals (SDG) from the review, analysis, and relationship of the different specific goals. It is specifically proposed to analyze the possible relationships of the new pedagogical models in PE as an engine of methodological change, with the goals derived from the 2030 Agenda to lay the foundations for future research related to the work that can be carried out from educational centers, specifically PE for sustainable development becoming a priority objective of the new legislative frameworks in education.

For all these reasons, this research’s main objective was to analyze and select the specific goals of the SDGs that can be implemented in the field of PE. In addition, having clarified which specific goals can be worked on in PE, this study aims to relate these specific goals to the different models based on PE practices.

## 2. Systematic Review Used

For this study, a critical review has been used, as defined by Grant and Booth [19], with the main aim being the critical evaluation of all the existing literature aligned with the study’s objectives, beyond mere description, including the degree, analysis, and conceptual innovation that it entails, in addition to identifying the most important elements in the field of knowledge. This approach to the study leads us to a future line of research or establishing a proposal or intervention model based on pedagogical models in our study, as is typical of this type of work as indicated by the authors mentioned above. Similarly, we could identify common elements with a narrative review, such as the search or review of the complete literature or not; and may or may not include a quality assessment, which is in line with our work. This type of review is truly useful in education since it allows us to detail in a justified and understandable way certain realities that may go unnoticed [20]. Narrative reviews help the reader understand the essence of the content displayed, providing evidence and justification for the text [21]. Its use in research is necessary and fundamental since it allows justifying facts, situations, and evidence from an explanatory point of view, and it is not new in the science and education of sport in our field [22].

In this case, the contribution is significant within PE, giving rigor to such substantial elements as methodology and evaluation.

Therefore, it is not a traditional systematic review, but rather an argumentative, critical explanation about the justification of the close relationships and the contribution from the PE area and specifically from pedagogical models that can be made to sustainable development as a priority objective globally. For this, the United Nations’ objectives under the concept of “Agenda 2030” and the specific goals derived from them are presented as the main elements, reflecting all the possible actions that can be carried out from the scope of PE. Therefore, it does not make sense to present the aspects related to the inclusion and exclusion criteria or the reliability of the data extraction, rather it is more related to systematic reviews of an analytical and quantitative nature.

## 3. Physical Education and Targets of the Sustainable Development Goals. A Proposal Selection

This critical review’s first objective was to select those SDG targets that could be implemented in PE. For this purpose, an exhaustive analysis of the institutional contributions is presented in Table 1. This table shows that of the 169 specific goals proposed by UNESCO, the relationship between sport and sustainable development can be seen in 68 of them, approximately 40% in the case of the Ibero-American Sports Council (IBC) [17]. However, the Sixth International Conference of Ministers and Senior Officials Responsible for Physical Education and Sport [15] selected a total of seven SDGs that relate to PA, sport, and PE. 

The importance of this relational analysis between PE and SDGs could be justified by the very significance UNESCO accords to PE. An example of this is to see how since the International Charter of Physical Education and Sport [23] in 1978, the role of sport and PE has been extolled as a fundamental right for everyone. Additionally, Agenda 2030 recognizes sport as an “important facilitator of sustainable development and peace”, adding that it can “increasingly contribute to making development and peace a reality by promoting tolerance and respect”, to “support the empowerment of women and youth, individuals and communities” or to “achieve goals in health, education and social inclusion” [24]. Despite this, analyzing the United Nations (UN) document for the ESD, PE, activity, or physical exercise is not mentioned [12]. This lack of precision in the role of PE within SDGs may carry some risk, going unnoticed by the relevant bodies. Despite institutional efforts to increase the number of PA practice hours, among other issues, the WHO recently showed that 80% of adolescents and young people still do not carry out the minimum recommended amount, and something that is extremely worrying is the increase in sedentary lifestyles [25]. This recent report has established new PA recommendations of approximately 60 min per day of moderate to vigorous aerobic activity for children and adolescents and at least 150 to 300 min per week for adults. In addition, during the COVID-19 pandemic period, the importance of performing PA routines to enhance the immune system has been highlighted [26].

The institutional analysis carried out through the proposals of the IBC (2019), the MINEPS (2017), and the Commonwealth (2017) was made considering the concept of Sport, Physical Exercise, and PA. Although a terminological differentiation is proposed in the Commonwealth case, it is not differentiated in the relationship with the SDGs. However, it is essential to note that these concepts are not synonymous and have specific limitations and possibilities independently. For this reason, to respond to the objective of this work, we have taken as a reference the indications presented by the institutions (Table 1) and thus was able to carry out an analysis exclusively from the exclusive concept of PE, since to the best of our knowledge, no document defines the role of PE in the contribution of UN SDGs. Therefore, PE may not have as many possibilities of intervention in some of the targets of the SDGs, or, on the contrary, it may be an essential piece in the achievement of some Agenda 2030 goals. Thus, it has been considered necessary to isolate the concept of PE and present a proposal where this matter can be related to the SDGs and their specific targets.

### 3.1. SDG 3. Good Health and Well-Being

Firstly, the analyzed document coincides with SDG 1 “no poverty” and 2 “zero hunger”, therefore the first relationship is established with SDG 3. Table 1 showed four SDG 3 that could be related to PE. However, it should be considered that target 3.3 cannot be directly treated in this area. On the other hand, both targets 3.4, 3.5, and 3.6 could be worked directly in PE. The improvement of mental health and well-being expressed in goal 3.4 is one of the most evident relationships established through PE sessions. There is enough evidence to confirm that PA practice increases students’ psychological quality [27,28], with it understood that such practice involves the activity carried out continuously and systematically. In relation to target 3.5, a direct relationship can be established with PE, as this subject can keep young people away from substance use [29,30]. Regarding sex education, as set out in SDG 3.7, a direct link should be considered as PE helps with the knowledge of one’s own body and strengthens sexuality [31]. Finally, SDG 3.6 has been added since driver education is a topic that appears in some PE curriculum, so it is feasible to consider a direct relationship between both. 

It is interesting to note that taking the name of SDG 3, health and wellbeing, as an exclusive reference, it could be understood that it is one of the SDGs most related to PE. However, out of the 13 incorporated targets, only four are considered for application through PE (31%).

### 3.2. SDG 4. Quality Education

The selection of goals made by the institutions studied makes SDG 4 one of the most relevant to PE. Firstly, target 4.1 is considered to have a direct relationship with PE as the UN has established PE as a “fundamental right of all” [23] and, therefore, it is a crucial component of equitable and quality education. Furthermore, there is sufficient evidence at the neuroscientific level that establishes a positive relationship between the realization of PA and academic and/or cognitive performance [32,33,34]. For example, improved concentration [35], better performance in general executive function [33,36], or an increased sense of well-being or dream quality [37,38]. Target 4.4 is oriented in the same direction by sport in general [14,17], as it involves developing personal skills related to employment [39]. Target 4.5 points out the importance of reducing inequalities, highlighting gender and vulnerable people. In this case, the relationship with PE would also be direct as it allows the integration of values such as teamwork, companionship, cooperation, and an ideal opportunity to develop co-education [40]. 

A similar situation occurs with target 4.7, which emphasizes the importance of improving knowledge to promote sustainable development. In this case, PE is working on PA implementation in natural environments and presenting sustainable alternatives such as the use of bicycles [41,42]. The IBC [17] established a two-way relationship with this goal. PE should be considered as an opportunity to generate self-constructed materials or the design of alternative sports that reuse a sports space or facility for a purpose other than its original one [43]. However, PE in itself cannot directly offer the improvement of a sports facility, so the relationship with this goal is indirect. Finally, the possibility of administering target 4.b to facilitate the increase of scholarships in developing countries has been discussed, but this possibility seems more the responsibility of sports institutions than schools.

### 3.3. SDG 5. Gender Equality

Gender equality in SDGs has been presented as an essential foundation for building a peaceful, prosperous, and sustainable world [1]. Specifically, in the case of PE, it has been described as a reflection of society, where gender norms are expressed in such a way that women are less likely than men to participate in PA outside of the educational field [15], and for this reason, PE should be an opportunity to promote the empowerment and leadership of women thus avoiding discriminatory stereotypes [17]. 

The analysis of institutions’ targets considered the direct relationship between PE and target 4.1 in eliminating discrimination against women. For example, the tone used in class to talk to students may be perceived as different amongst genders [40]. Factors such as these need to be monitored during PE classes to generate student awareness of gender inequalities. Likewise, PE in pre-school education or the first years of secondary education is an opportunity to show that there are no differences in physical performance between men and women [41], this justification being extrapolated to goals 5.2 and 5.5. Furthermore, as stated by MINEPS [15], “*the strong and active participation of women in decision-making processes has a major impact on social development*”. (p. 11), so the empowerment of women (target 5.c) can be achieved through the presentation of physical-sports initiatives in PE that are attractive to them, but these initiatives must also have the support of institutions outside the education sector to offer an opportunity for practice outside the educational context.

Finally, the inclusion of target 5.3 is not considered for applicability to PE. However, the suggestion of an indirect relationship is proposed by the Commonwealth and is justified by the empowerment that sport can generate in women [14]. 

### 3.4. SDG 8. Decent Work and Economic Growth

This SDG aims to ensure inclusive and sustained economic growth that can generate decent jobs for all [1]. Although the sport has been described as a source of economic growth and an opportunity for decent employment [17], the reality is that PE has minimal direct relationship with this SDG. However, the values worked on in PE are similar to skills that are supposed to be crucial to employability, such as cooperation, fair play, or goal management [14,39].

However, from a perspective focused exclusively on job creation, PE does not have a clear relationship, although it is true that a competitive use of the PE through, for example, the Sports Education model could allow students to learn sport-related professions such as analyst, trainer or journalist among others [44] it cannot be considered a direct relationship between employability and the PE.

This lack of a direct relationship with the PE has meant that the IBC [17] has not established a relationship with any of the goals among the institutional ones analyzed. Of the purposes that have been related, 8.3 and 8.9 are considered to have the potential to be worked on in PE. The first of these emphasizes the possibilities of sport in general as a way to entrepreneurship and innovation, although this goal could also be related to the 4.4. As mentioned above, among the PE pedagogical models, the Sports Education model can be a method to promote employment alternatives directly related to sport. As highlighted in MINEPS [15], “*The attractiveness of sport for young people makes it a valuable framework for employability initiatives*” (p. 10). In addition, target 8.9 highlights the opportunity to generate sustainable tourism while promoting local culture. PE has a direct relationship with this goal. It works on content that supports alternative employment in the framework of natural environments and sustainability, and the work of traditional sports and games or local food products is also related to the PE.

### 3.5. SDG 10. Reduced Inequalities

SDG 10 aims to reduce inequalities to ensure the Sustainable Development Goals [1]. Sport is undoubtedly an opportunity for social inclusion and diversity, as equal opportunities can be promoted through sport [17]. Targets 10.2 and 10.3 include the importance of this equality, which PE can work on directly, as it has been shown that PE sessions promote cooperative attitudes and inclusion [45,46,47]. Regarding goal 10.7, the contribution of PE is not considered, as both institutions [14,15] selected this goal considering the benefits of professional sport to promote opportunities in underdeveloped countries. This aspect does not apply to PE.

### 3.6. SDG 11. Sustainable Cities and Communities

The IBC [17] relates SDG 11 mainly through the bidirectionality of accessible sports facilities in cities and the opportunity for better social cohesion and equal opportunity. Therefore, although the proposal of goals made by the three institutions analyzed is coincidental (11.3 and 11.7), in both cases, their relationship with PE is not contemplated. A relationship with PE could be considered, for example, through activities framed within a service-learning aimed at improving or developing the care of a park to carry out PA. However, this is a characteristic of the service-learning model rather than PE itself.

### 3.7. SDG 12. Responsible Consumption and Production

This SDG relates to the responsible use of natural resources to avoid destructive effects on the planet [1]. This approach is mainly related to institutionalized sports-related properties; as the IBC [17] indicates, sport can improve sustainable living awareness, both for products and sports facilities or events. From an educational perspective, MINEPS [15] focuses on the importance of educational programs to raise awareness and thus influence attitudes to change consumer behavior and the real resources (targets 12.2, 12.5, and 12.8).

From the perspective of PE, an indirect relationship with the goals that compose SDG 12 could be considered. For example, the realization of educational activities and projects that aim to explain the cost of natural resources entails the disposal of sports materials. In addition, describing the environmental impact that PA can cause in a natural environment (plastic bottles, packaging, etc.) could be a strategy to consider for responsible consumer awareness. However, these goals may not be regarded as the direct responsibility of PE, rather the education system’s responsibility in general.

### 3.8. SDG 13. Climate Action 

The environmental problems suffered by humanity today have not only been collected in an SDG but are also dealt with in SDG 14 (underwater life) and SDG 15 (life terrestrial ecosystems). Specifically, the analyses performed have pointed exclusively to target 13.1 and its relationship with sport and PA [14,15]. The IBC does not relate to any of these SDG targets. Despite this, this institution points out how sports culture usually promotes environmental care in any media since it is a useful tool for educating and raising awareness among young people [17]. In addition, MINEPS [15] emphasizes the value of sport in building resilience and the capacity to adapt to risks related to climate and natural disasters (target 13.1). However, none of the approaches directly highlights the value of PE in working on this SDG. 

In the proposal presented, the PE possibilities for environmental care are considered since it is one of the main contents. For example, the direct relationship with target 13.3, which specifies the importance of education and awareness to avoid environmental problems, should be assessed. Some research has also shown PE students’ opinions when practicing sports such as plogging, where they jog through different environments while collecting polluting waste [48]. Finally, institutions should consider reviewing SDGs 14 and 15 to analyze a potential relationship between sports and PE.

### 3.9. SDG 16. Peace, Justice, and Strong Institutions

Conflict, insecurity, or injustice are a severe threat to sustainable development [1]. The IBC [17] details that sport and PA can help reduce violence, improve unity, as well as promote dialogue and social cohesion. 

Therefore, the institutional analysis carried out shows that six of the targets proposed in this SDG have been generally related to sport, PE, and PA. Despite this, it can be seen that this SDG refers mainly to improving peace and justice in official organizations and institutions, and, therefore, is far from the potential influence of PE, assuming that most of the targets do not have a clear relationship with the subject of PE. While it is true that the practice of PE within the framework of activities that require cooperation and collaboration, such as sports, would help improve interpersonal relations among students [39], an indirect relationship could be established with goal 16.7. 

In addition, this goal could also be related to applying the Personal and Social Responsibility Model, which has been considered a valid pedagogical model in PE for improving citizenship [49,50]. Finally, the practice of sports governed by rules can explain the importance of respecting operational standards in a community.

### 3.10. SDG 17. Partnership for the Goals

Finally, SDG 17 considers the importance of global cooperation and partnerships to achieve all the targets set in Agenda 2030 [1]. Undoubtedly, concerning the values that sport or PE represents, it can help contribute to emotional connections and encourage cooperative habits [17]. However, as in SDG 16, the specific analysis of the targets shows that this cooperation is mainly aimed at global institutions generating synergies that help achieve the SDGs, especially in developing countries. Therefore, although PE is a great tool to promote cooperation among equals, its influence would be far removed the targets proposed by SDG 17.

As shown in Section 3, a new proposal of specific targets is suggested for PE by way of synthesis. From SDG 3, target 3.3 related to communicable disease transmission is proposed to be eliminated, and target 3.6 is integrated because road safety is a content that can be addressed in PE. The rest of the proposed targets (3.4, 3.5, and 3.6) are maintained. SDG4 stays almost in its entirety except for target 4.b related to the assignment of scholarships to developing countries. A similar circumstance occurs with SDG5 since the proposed list of targets is accepted, except for target 5.3, which aims to avoid harmful practices such as child marriage. However, SDG8 has been limited since only two targets consider a relationship with the PE (8.3 and 8.9). In SDG10, the decision was made to eliminate target 10.7, which is dedicated to facilitating migration and mobility. For SDG 12, five of the six targets proposed remain (except 12.6). For SDG 13, the proposed goal is accepted, and it is suggested that 13.3 be added, which seeks to improve education for climate change. Concerning SDG 16, despite offering six proposed targets, 16.7 is the one that has been suggested with the potential to be worked on in PE. Finally, SDG 11 and 17 have been eliminated because their goals have not been found to have transferable aspects to PE.

## 4. Practice-Based Models, Contents, and SDGs. Working in Physical Education 

Now that the first objective has been achieved, this critical review’s second objective was to relate the specific goals of the SDGs to different practice-based models. Achieving this objective is key because PE is characterized as a subject that offers a great methodological variety due to its content nature. This diversity of methodology leads to the appearance of different teaching models that have demonstrated their validity in PE. These models can be classified as Cooperative Learning (CL), Sports Education Model (SE), or the model of Personal and Social Responsibility (PSR). Additionally, in recent years, other emerging options have appeared such as Adventure Education (AE), Attitudinal Style (EA), Health Education (HE), or Self-construction of materials (SC).

### 4.1. Cooperative Learning

The approach to cooperative learning (CL) techniques from PE will allow personal skills that improve positive interdependence, social and group responsibilities, individual responsibilities and promote interaction [45,51,52]. Although CL techniques are not exclusively characteristic of PE, the nature of PE implies greater contact and interaction which means that the benefits of cooperation are strengthened [51,53].

Cooperative performance in PE could help contribute to the achievement of different goals. A clear relationship can be observed with target 16.7, which aims to guarantee inclusive, participatory, and representative decisions, which are characteristics that are intrinsic and specific to CL [52,53]. Another example occurs with target 4.4, which deals with developing entrepreneurial competencies to facilitate employment access, with CL leading to developing important innovative and cooperative attitudes in students for job demands [54,55]. In addition, target 4.5, which focuses on promoting equality for vulnerable people, can be worked on from a CL perspective thanks to the fact that all participants in a cooperative group must value their colleagues’ work to achieve a common goal [56,57]. Target 8.3 could be added to these suggestions since it also considers entrepreneurship, creativity, and innovation in the business context, where the social and individual skills of PE acquire special significance [58,59].

### 4.2. Model of Personal and Social Responsibility

The Personal and Social Responsibility (PSR) model has been shown to be a valid pedagogical model in PE to develop students’ competencies related to individual and group responsibility [50,60]. Therefore, this model encourages respect, equality, and social values [47,61] to be connected to those SDG goals with similar objectives.

For example, social responsibility has been documented as a model that allows social awareness development regardless of gender [62]. Therefore, targets 5.1, 5.2, 5.5, and 5.c together seek to eliminate inequalities between men and women, and at the same time, promote and favor the empowerment of women and equal opportunities. Additionally, as was the case with CL, PSR contributes in a direct way to develop people’s personal and social awareness [63], so it could also be related to the achievement of target 16.7 to guarantee inclusive decisions and participation, in addition to the goals proposed in SDG 10, such as 10.2 based on the social, economic and political inclusion of all people, and 10.3 that aims to guarantee equal opportunities and reduce inequalities [60,61]. 

### 4.3. Sports Education Model

Since this model’s main objective is to create a context that generates sports experiences as authentically as possible [44], it could be interesting to link it with those SDGs related more to employability. It must be borne in mind that sports, directly and indirectly, produce a significant amount of money and employment. For example, according to INE, sports in Spain account for 3.3% of GDP and more than 400,000 jobs, representing 2.1% of total employment [64]. In Europe, according to European Union data, it represents 2.12% of GDP and 2.72% of jobs, and at a global level, 600,000 million a year, or 1.5–2% of GDP worldwide [65].

One of the excellent characteristics of this model is the variety of roles it offers to students beyond the role of an athlete. For example, the role of coach, physical trainer, journalist, marketing or different management positions, mediators, risk control, etc., can be implemented [44]. In addition to this multitude of functions, it is, of course, necessary to take into account the social and personal skills that this model entails, such as teamwork, the transfer of responsibilities, empathy, and the promotion of autonomy [66].

Due to all these factors, the SDGs’ goals could be related to this pedagogical model. For example, two goals of SDG8, i.e., 8.3 on fostering entrepreneurship, creativity, and innovation, and 8.2, focused on achieving higher levels of economic productivity through diversification, technological modernization, and innovation. In addition, target 4.4 aimed at improving skills to access employment, decent work, and entrepreneurship. Finally, it should be noted that all those goals related to the development of socialization or respect could also be worked on through this model; for example, target 16.7 seeks to guarantee inclusive, participatory, and representative decisions that respond to needs.

### 4.4. Adventure Education Model

This model was created to generate a learning context where interaction with the natural environment involves a series of elements of natural or fictitious risks [67]. This model has been related to green pedagogies, which involve a work philosophy that consists of the concept of a human being with an innate capacity to develop its full potential in interaction with the natural environment. In addition, Gehris et al. [68] pointed out that this model helps promote activities in natural environments in students’ free time. In addition, the WHO points to the practice of PA as an opportunity to stimulate care for the environment [25] Therefore, it seems to indicate that the realization of PA in natural surroundings promotes environmental care; thus, a relationship could be established between some of the Adventure Education (AE), and different SDG goals.

Although caring for the environment is included in three SDGs (13, 14, and 15), none of the reference institutions chose to relate to SDG 14 and 15. Therefore, from the proposed goals presented in this work, the relationship of AE with goals 13.1, to strengthen the capacity to adapt to risks related to climate and natural disasters in all countries and 13.3 to improve education, awareness, and human and institutional capacity regarding the mitigation of climate change should be considered. Additionally, the proposed goal 12.1 should be considered since it aims to manage natural resources sustainably and efficiently. Finally, goal 8.3 emphasizes the development of sustainable tourism that helps promote local culture, so it has been presented, as AE could contribute to this goal thanks to the promotion of PA in the natural environment with all that it entails.

### 4.5. Self-Construction of Materials

The self-construction of materials has been a common way of working in PE for years, having, among other objectives, to develop an ecological conscience both in PE students and in the educational community [43]. In addition, the self-construction process of material has been documented as an ideal tool to develop the cooperation capacity and creativity of students [43], aspects highlighted in different goals of the SDGs.

For this reason, this emerging model could be related to the goals of SDG 13 (13.1 and 13.3) that generally defend the importance of respect for the environment or the reduction of the consequences of global warming. Additionally, the relationship of the materials self-construction model with SDG 12 and its targets 12.1 regarding sustainable consumption and production, 12.2 on the efficient use of natural resources, 12.5 about the reduction of waste generation, and 12.8 to guarantee quality information is direct. 

Finally, the cooperative environment that is generated during the creation of a self-constructed material, as well as the creativity that this act entails, will enable the development of skills related to entrepreneurship (targets 4.4 and 8.3).

### 4.6. Health Education

Haerens et al., 2011 [69] pointed out the importance of conceiving PE not only as the development of a set of skills but that health should be at the center of the approach to the subject. Although this objective can be understood as basic or general to PE, this model focuses on the effectiveness of achieving this and not just the motor aspects themselves. In this way, it will be possible to obtain greater regulation of PA outside the educational context. That is why Haerenes et al. [69] highlighted the importance of conceiving this model with the idea of health education (HE) being a “lifelong learning”.

Therefore, HE must be related to the goals that appear in SDG3-Health and Well-being, specifically target 3.4 on the reduction of premature mortality and promotion of mental health and well-being and target 3.5 on the reduction of addictive substance abuse. The positive effects of PE and PA on young people are well known because of all the strategies aimed at promoting healthy habits, as it is understood that as a habit or routine that is repeated regularly, an active occupation of free time will generate more vital, happier citizens with a higher quality of life.

### 4.7. Beyond the Models, the Contents of PE

In addition, to the relationship between the proposed models, the nature of PE and the contents that are usually covered in its curriculum allows the development of different goals that have been selected in this proposal. For example, road safety education represented in target 3.6 has been included in some physical education curricula. This promotes the use of bicycles among students to contribute to sustainable and healthy mobility. On the other hand, although it is not included in any goal, it does speak in a general way in SDG-4 of quality education. In this sense, it could be interesting to relate the neurocognitive and academic benefits of performing PA in a chronic [69,70] or acute way, for example, using active breaks as an educational tool [71,72].

In summary, for these models, it should be considered that not all of them will be able to contribute to obtaining the SDGs. However, the selection of goals that has been carried out has been regarded as the content that the PE can work on, allowing a more significant contribution to the goals in the selected SDGs. For all these reasons, Table 2, presented below, shows a synthesis of the 24 goals that can be worked on from the PE perspective and the relationship with the different models-based practice basic pedagogy and the contents of the subjects. 

Therefore, the practical applications of the different contents worked on in PE and the models-based practice could be contributed to the specific goals of the 2030 Agenda directly and indirectly. For example, the development of environmental awareness and the sustainable use of resources through an adventure education model or the self-construction of materials; the development of virtues of cooperation, teamwork, or respect thanks to the cooperative learning models and that of personal and social responsibility; work performance and entrepreneurship in an indirect way through the sports education model; or simply improving adherence to performing PA thanks to the health education model. This critical review will allow for action strategies to develop the SDGs in an educational context. It may also contribute to transferring these actions beyond the educational community (e.g., service-learning), and initial and permanent training developed as an engine of change. These aspects and those that go through defining realistic objectives and establishing verifiable results, indicators, and sources of data, as well as ensuring that the objectives are in accordance with the available resources and develop a culture of institutional capacity to achieve said goals, will achieve greater outcomes. Having a reliable how, where, and why to invest resources to maximize contributions to the development goals in a more practical sense.

## 5. Conclusions

PE is a transcendental subject that could contribute to achieving the goals and objectives set out in the 2030 Agenda. Beyond the healthy virtues of PA and exercise, PE creates a context very favorable that allows the development of cooperation, respect, coeducation and entrepreneurship, all aspects related to the development of the SDGs. However, regarding the first objective proposed in this research, it can be concluded that not all the SDGs’ goals can be implemented in PE. After our analysis, of the 169 goals proposed by UNESCO, only 24 can be developed in PE classes. In relation to these 24 goals, and in responding to the second objective of the research, it has been shown that most of them can be worked through in the context of PE. In addition, some practice-based models such as cooperative learning, the model of personal and social responsibility, or the self-construction of materials will allow the development of more sustainable behaviors in young students.

However, we believe that future research projects should be developed to facilitate the application of the SDGs in the educational context. For example, improving the ability to measure and evaluate the contribution of sport, PE, and PA to the SDGs will be key to ensuring that they occur. In relation to this idea, the Commonwealth, through UNESCO, published a practical guide and indicators to measure the contribution of sport, PE, and PA in achieving the SDGs. A similar analysis focused exclusively on PE will allow teachers to establish more stable criteria to develop the goals of the 2030 Agenda in a more specific way in the educational arena. Simultaneously, it will make it possible to evaluate the degree of achievement of said goals, so it could be interesting to establish curricular links between the 17 SDGs and the different academic subjects. This data will allow more reliable conclusions to be reached on how, where, and why to invest to maximize the contribution to development goals in a more practical sense.

## Figures and Tables

**Table 1:** Relationship of Physical Education, Sport and Physical Activity with Sustainable Development Goals and their specific targets according to institutional analysis.

Sustainable Development Goals	Specific Target	IBC [17]	MINEPS [15]	Commonwealth [14]
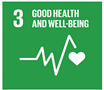	**Sustainable Development Goals 3.** *Good health and well-being*	2	4	4
	3.3 Ending epidemics, waterborne, and other communicable diseases	-	✓	+
	3.4 Reduction of premature mortality and promotion of mental health and well-being	+	✓	✓
	3.5 Substance Abuse Reduction	+	✓	+
	3.7 Ensure universal access to sexual and reproductive health services	-	✓	+
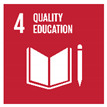	**Sustainable Development Goals 4.** *Quality education*	5	4	6
	4.1 Ensure that all girls and boys complete primary and secondary education, which should be free, equitable, and of good quality	-	✓	+
	4.4 Improving skills for access to employment, decent work, and entrepreneurship	+	✓	✓
	4.5 Reduction of gender disparities in education and equality of vulnerable people	+	✓	✓
	4.7 Improving knowledge to promote sustainable development (e.g., sustainable lifestyles)	✓	✓	✓
	4.a Improvement of School Facilities	✓	-	✓
	4.b Increasing the number of scholarships available to developing countries	✓	-	✓
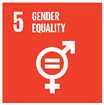	**Sustainable Development Goals 5.** *Gender equality*	3	4	5
	5.1 Elimination of discrimination against all women and girls	+	✓	✓
	5.2 Eliminate all forms of violence against all women and girls in the public and private spheres	-	✓	+
	5.3 Eliminate all harmful practices, such as child, early and forced marriage	-	✓	+
	5.5 Women’s participation and equal opportunities	+	✓	✓
	5.c Promoting gender equality and empowerment of women and girls		-	✓
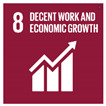	**Sustainable Development Goals 8.** *Decent work and economic growth*		5	7
	8.1 Maintain economic growth per capita in accordance with national circumstances	-	-	✓
	8.2 Achieve higher levels of economic productivity through diversification, technological upgrading, and innovation, including a focus on high value-added and labor-intensive sectors	-	✓	+
	8.3 Entrepreneurship, creativity and innovation, and promoting the formalization and growth of enterprises	-	✓	+
	8.5 Achieve full and productive employment and decent work for all women and men, including young people and persons with disabilities, and equal pay for work of equal value	-	✓	+
	8.6 Significantly reduce the proportion of young people who are not employed and are not in school or training	-	✓	✓
	8.7 Ending contemporary forms of slavery and eliminating child labor	-	✓	+
	8.9 Promote sustainable tourism that creates jobs and promotes local culture and products	-	-	+
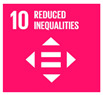	**Sustainable Development Goals 10.** *Reduced inequalities*	2	2	2
	10.2 Social, economic and political inclusion of all people	+	✓	✓
	10.3 Ensuring equal opportunities and reducing inequality of outcomes	+	-	-
	10.7 Facilitate orderly, safe, regular, and responsible migration and mobility of people.	-	✓	+
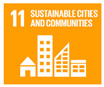	**Sustainable Development Goals 11.** *Sustainable cities and communities*	2	2	2
	11.3 Inclusive and sustainable urbanization	✓	✓	+
	11.7 Universal access to safe, inclusive, and accessible green spaces and public spaces	✓	✓	✓
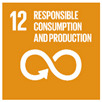	**Sustainable Development Goals 12.** *Responsible consumption and production*	2	5	5
	12.1 Sustainable consumption and production	-	✓	+
	12.2 Sustainable management and efficient use of natural resources	-	✓	+
	12.5 Significantly reduce waste generation	-	✓	+
	12.6 Adoption of sustainable practices in companies	✓	✓	✓
	12.8 Ensuring information and knowledge relevant to sustainable development	✓	✓	✓
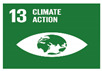	**Sustainable Development Goals 13.** *Climate action*		1	1
	13.1 Strengthen capacity to adapt to climate-related risks and natural disasters in all countries	-	✓	✓
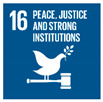	**Sustainable Development Goals 16.** *Peace, justices and strong institutions*	2	5	6
	16.1 Reduction of all forms of violence and related mortality rates worldwide	✓	✓	+
	16.2 Ending abuse, exploitation, trafficking, and all forms of violence and torture against children	-	✓	✓
	16.4 Fight against all forms of organized crime	-	✓	✓
	16.5 Significantly reduce corruption and bribery in all its forms	✓	✓	+
	16.6 Create effective and transparent institutions at all levels that are accountable	-	✓	✓
	16.7 Ensure inclusive, participatory, and representative decisions that respond to the needs	-	-	+
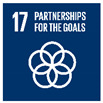	**Sustainable Development Goals 17.** *Partnerships for the goals*	2		4
	17.3 Mobilizing additional financial resources for developing countries from multiple sources	-	-	✓
	17.9 Increase effective and targeted capacity building activities in developing countries	-	-	✓
	17.16 Promoting effective partnerships in the public, public-private, and civil society spheres	✓	-	+
	17.17 Promoting effective partnerships in the public, public-private, and civil society spheres	-	-	+
	17.19 Development of indicators to measure progress in sustainable development	✓	-	-

Note. IBC: Ibero-American Sports Council; MINEPS: Ministers and Senior Officials Responsible for Physical Education and Sport; ✓ (Direct and bidirectional relationship); + (Indirect or unidirectional relationship); - (No relationship specified).

**Table 2:** Proposed relationship between Models-Based Practice and the specific targets of the SDGs suggested for Physical Education.

Specific Targets Suggested	Models-Based Practice						
	CC	CL	PSR	SE	AE	SC	HE
3.4 Reducing premature mortality and promoting mental health and well-being	✓						✓
3.5 Reduction of substance abuse	✓						
3.7 Ensuring universal access to sexual and reproductive health services	✓						
3.6 Halve the number of deaths and injuries caused by road traffic accidents worldwide	✓						
4.1 Ensure that all girls and boys complete primary and secondary education, which should be free, equitable, and of good quality	✓	✓					
4.4 Improving skills for access to employment, decent work, and entrepreneurship		✓	✓	✓			
4.5 Reduction of gender disparities in education and equality of vulnerable people	✓	✓	✓				
4.7 Improving knowledge to promote sustainable development (e.g., sustainable lifestyles)					✓	✓	
4.a Improvement of school facilities					✓		
5.1 Elimination of discrimination against all women and girls	✓	✓	✓				
5.2 Eliminate all forms of violence against all women and girls in the public and private spheres	✓	✓	✓				
5.5 Women’s participation and equal opportunities	✓	✓	✓				
5.c Promoting gender equality and empowerment of women and girls	✓	✓	✓				
8.3 Entrepreneurship, creativity and innovation, and promoting the formalization and growth of enterprises				✓		✓	
8.9 Promote sustainable tourism that creates jobs and promotes local culture and products					✓		
10.2 Social, economic and political inclusion of all people		✓	✓				
10.3 Ensuring equal opportunities and reducing inequality of outcomes	✓	✓	✓				
12.1 Sustainable Consumption and Production					✓		
12.2 Sustainable management and efficient use of natural resources					✓	✓	
12.5 Significantly reduce waste generation						✓	
12.8 Ensure information and knowledge relevant to sustainable development						✓	
13.1 Strengthen capacity to adapt to climate and natural disaster-related risks in all countries					✓	✓	
13.3 Improve education, awareness, and human and institutional capacity for climate change mitigation, adaptation, mitigation, and early warning					✓		
16.7 Ensure inclusive, participatory, and representative decisions that respond to the needs	✓	✓	✓				

CC, Content of curriculum CL, Cooperative Learning PSR, Personal and Social Responsibility Model SE, Sports Education AE, Adventure Education SC, Self-Construction material HE, Health Education, ✓, Potential relationship.
